# Differentiation approach in education: Tailoring instruction for diverse learner needs

**DOI:** 10.1016/j.mex.2025.103163

**Published:** 2025-01-07

**Authors:** Nigora Goyibova, Narzulla Muslimov, Gulnoza Sabirova, Nargiza Kadirova, Barnoxon Samatova

**Affiliations:** a “Tashkent Institute of Irrigation and Agricultural Mechanization Engineers”, National Research University, Kari Niyaziy 39 str, Tashkent, 100000, Uzbekistan; b Tashkent State Pedagogical University, Bratislava str, Tashkent, 100000, Uzbekistan

**Keywords:** Differentiation, Differentiation learning, Differentiation approach, Multiple intelligence, Differentiated instruction, Diverse learning

## Abstract

**Background:**

In education, differentiation creates engaging and challenging experiences that improve engagement and accomplishment by customizing instruction, content, and assessment to match the needs of varied learners.

**Method:**

This study examined differentiation in education across diverse national contexts, exploring strategies like task complexity differentiation, flexible grouping, and multiple intelligences, supplemented by case studies in foreign language classrooms to observe teacher adaptations for individual needs.

**Result:**

While case studies demonstrated that successful implementation improves academic performance, motivation, and engagement, especially in foreign language teaching, the analysis showed that differentiation is typically framed through student-focused or teaching methods, underscoring the need for system-oriented research.

**Conclusion:**

In order to address the varying needs of students, foster inclusion, and improve academic performance, differentiation in education is crucial. To develop supportive learning environments that play to each student's individual strengths, it takes system-wide research, teacher training, and resources.

## Review Highlights

• By adapting instruction, content, and assessment to meet the needs of various learners, differentiation in education improves student engagement and accomplishment.
• Differentiation addresses students' varied learning styles and strengths by utilizing the many intelligences theory to customize instruction, content, and evaluation.
• The study underscores the importance of teacher training and system-wide research to effectively integrate multiple intelligences into educational practices, fostering inclusivity and personalized learning.

## Graphical abstract

**Figure:**
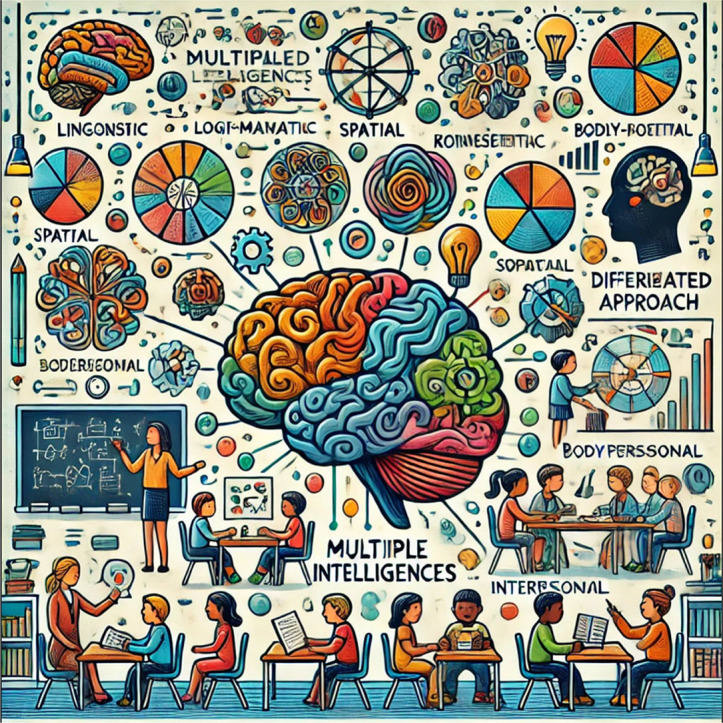

Specifications table

**Table:** 

Subject area:	Psychology
More specific subject area:	Multiple intelligence of differentiation approach
Name of the reviewed methodology:	Differentiation approach
Keywords:	differentiation, differentiation learning, differentiation approach, multiple intelligence, differentiated instruction, diverse learning
Resource availability:	
Review question:	What is the difference of differentiation approach and differentiated learning?

## Background

Differentiation recognizes that students have varying proficiency levels in writing and may require different strategies and support to improve [1]

### What is differentiation?

The root of “differentiation” is “differentiate,” which comes from the Latin word “differentia,” meaning “difference” or “distinction.” In its original sense, “differentiate” refers to making or becoming different or distinct. According to the Cambridge Dictionary, “differentiation” is the process or fact of being different or creating something different from other similar things. (*Cambridge Online Dictionary*).

**Differentiation of learning** - this is a grouping of students based on considering their individual abilities for training in slightly different curricula, programs, and technologies [2].

**Differentiation approach** - groups are made based on member traits, using various methods and strategies to support teaching in groups with similar abilities. This principle aims to enhance teaching effectiveness by considering students' unique characteristics like interests, creativity, and learning abilities, guiding the selection and adaptation of educational goals, content, and teaching methods accordingly [3].

Educational policies in various countries mandate that teachers tailor their lessons to meet diverse student needs [4]. Differentiated instruction is where teachers modify their lessons to accommodate each child's unique needs, including prior knowledge and skills, interests, and classroom behavior [5]. To proactively adjust their lessons, teachers should consistently monitor and assess their students' needs, making deliberate decisions based on these evaluations during lesson planning and implementation [6]. Differentiated instruction is often defined as an adaptive and proactive educational approach (Prast, E. J et al.) . According to Jager, implementing adaptivity and proactivity necessitates multiple perspectives: understanding teachers' thought processes during lesson planning is essential to identify proactivity, and gathering information on students' experiences and changes in academic performance is crucial to determine if the adjustments effectively address students' needs [7]. Tomlinson [5] suggested that teachers can modify various aspects of a lesson to cater to students' needs. When adjusting the process, teachers offer varied learning activities or instructions, which involves tailoring the level of guidance during inquiry and design tasks to suit students' needs [8]. The proactive nature of differentiated instruction necessitates that teachers make numerous intentional decisions about their teaching [6] outlined a differentiation skills hierarchy and identified the chronological sequence of these decisions.

## Method details

### Literature review

The differentiation method in education is grounded in the understanding of students' diverse cognitive abilities and prior knowledge, requiring tailored instructional strategies that create supportive learning environments and accommodate individual needs, inspired by Vygotsky's theory and brain-based learning principles, with evidence from foreign language education research supporting its effectiveness. [9]. The differentiation approach in education plays a crucial role in enhancing student motivation. By recognizing and catering to individual differences in students' readiness, interests, and learning profiles, differentiation promotes a sense of relevance and engagement in the learning process. When students see that their individual needs and preferences are recognized through differentiated instruction, they are more likely to feel valued and motivated, especially when given choices that align with their interests and strengths, fostering a sense of ownership and enhancing intrinsic motivation [10].

In studying motivation, it is crucial to focus on students' needs, as understanding these needs helps shape the direction of their learning and mental state, driving activity, forming motives, and enabling goal-setting, which teachers can foster by adapting lessons to meet students' needs and setting collaborative goals to enhance the effectiveness of the educational process [11]. The differentiation approach in education can be closely connected with the theory of multiple intelligences proposed by Howard Gardner. Gardner's theory suggests that intelligence is not a singular, fixed entity but a combination of different types of intelligence, including linguistic, logical-mathematical, spatial, bodily-kinesthetic, musical, interpersonal, intrapersonal, and naturalistic intelligence. Each individual possesses a unique blend of these intelligences, with varying strengths and preferences [12]. When implementing the differentiation approach, educators recognize and value this diversity of intelligence among their students. They design instruction and learning experiences that cater to different types of intelligence, allowing students to engage with content in ways that align with their strengths and interests. For example, a teacher might provide opportunities for students to demonstrate their understanding of a concept through various modalities: *Linguistic learners* prefer writing essays or giving oral presentations. *Visual-spatial learners* might benefit from creating diagrams, charts, or drawings. *Kinesthetic learners* might excel in hands-on activities or experiments. *Musical learners* enjoy composing songs or rhythms related to the topic.

By integrating multiple intelligences into the differentiation approach, educators create a more inclusive and engaging learning environment where students can thrive based on their unique talents and abilities. This approach honors the diversity of learners and fosters a deeper understanding and appreciation of the subject matter [13]. The theory of multiple intelligences is a vital tool for educators across various disciplines. It allows them to transform the teaching-learning process and actively engage students through appropriate activities often missing in traditional school settings. The diversity among learners and high expectations for their learning makes teaching incredibly challenging. Addressing individual differences remains a significant challenge in education today, but it is also a crucial strategy for resolving many educational discrepancies. Gardner's theory of multiple intelligences has numerous implications for teachers, some of which are beyond their control.

Gardner's theory posits that autonomous powers and capabilities shape each person's intelligence level and that every child has potential intelligence in one or more areas. According to Gardner, intelligence is not limited to two regions but includes success in music, sports, art, self-awareness, and evaluation. He views intelligence as the ability to solve problems and achieve results within cultural contexts. Understanding the multiple intelligence profiles of secondary school students enables teachers to better know their students and understand their similarities and differences. Teachers should consider students' dominant intelligence areas when planning education and educational environments, encouraging proactive participation. Utilizing students' strengths positively impacts other areas. Research indicates that students' interests and intelligence areas vary by gender, and these natural differences must be considered. Addressing socio-economic diversity can prevent potential inequalities. Assigning tasks aligned with students' skills contributes to their personal development. Being the best judges of their abilities and differences, students should have their self-assessment results included in their personal development records [14].

According to Russell Jay Hendel, differentiated instruction (DI) boosts students' academic performance and satisfaction with learning, especially when teachers receive proper training beforehand. However, the vast amount of DI literature can overwhelm many educators, making it difficult for them to implement DI effectively for students with different learning styles. He proposes a transdisciplinary approach to bridge the gap between theory and the challenging practical application of DI. 1) Drawing from architecture, the concept of universal design is introduced, advocating for buildings to be initially constructed to accommodate everyone rather than trying to make adjustments after construction, which can be expensive and inefficient. 2) Neuroscience suggests that higher cognitive brain functions, like executive function, can be effectively addressed through a multi-modal approach, emphasizing the need for inclusive teaching methods that cater to various learning preferences. 3) Industrial psychology highlights the importance of goal-setting and breaking down complex tasks into manageable steps, promoting clear, achievable, and challenging objectives. 4) Combining goal-setting with the concept of self-efficacy from social psychology, utilizing software technology with differentiated difficulty levels is advocated, enabling students to assess and improve their learning through self-regulation. This transdisciplinary approach proposes several innovative strategies for DI, which it hopes other educators and researchers will explore further [15]. Drawing from the concept of universal design means creating learning environments and educational materials that are accessible and inclusive for all students, regardless of their abilities or backgrounds. This approach recognizes that students have diverse needs, learning styles, and abilities and aims to design educational experiences that cater to this diversity from the outset. For example, in the design of classrooms, universal design principles might involve: Ensuring classrooms are spacious and well-lit to accommodate students with mobility issues or visual impairments. We provide flexible seating arrangements for different learning preferences and physical needs. They are incorporating technology and multimedia resources that students with various learning styles and abilities can access. They offer materials in multiple formats (e.g., digital, audio, tactile) to accommodate different learning needs and preferences. Implementing inclusive teaching strategies that engage all students and provide opportunities for active participation.

By applying universal design principles in education, schools can create inclusive learning environments that benefit all students, regardless of their differences. This proactive approach avoids the need for costly and time-consuming adjustments or accommodations later on, leading to more efficient and effective educational experiences for everyone.

In education, insights from neuropsychology highlight the importance of considering higher cognitive brain functions, such as executive function, in designing instructional strategies. Executive function encompasses skills like planning, organization, and self-regulation, which are crucial for academic success.

A multimodal approach to teaching acknowledges that students have diverse learning preferences and strengths. Therefore, instruction should encompass various modalities, such as visual, auditory, kinesthetic, and tactile. This approach recognizes that different students learn best through different sensory channels and emphasizes the need for inclusive teaching methods that cater to these diverse learning preferences. For example, in a classroom using a multi-modal approach: Visual learners benefit from diagrams, charts, and graphic organizers. Auditory learners might excel with lectures, discussions, and audio recordings. Kinesthetic learners might thrive with hands-on activities, experiments, and role-playing exercises. Tactile learners might engage more effectively with tactile materials, manipulatives, and learning aids.

Educators can create inclusive learning environments that engage students of all learning preferences and abilities by incorporating a multi-modal approach into teaching practices. This approach promotes a more profound understanding, retention, and application of knowledge, ultimately leading to improved student academic outcomes. In education, integrating goal-setting with the concept of self-efficacy from social psychology emphasizes empowering students to control their learning processes. Self-efficacy refers to an individual's belief in their ability to succeed in specific tasks or situations. By combining goal-setting with self-efficacy, educators aim to enhance students' confidence in their skills and motivate them to pursue their learning goals. One way to implement this approach is by using software technology that offers differentiated difficulty levels. This means providing students with learning tasks or activities that vary in complexity and challenge, allowing them to select the level that matches their current skill level and learning pace. For example, a math software program might offer practice exercises ranging from essential addition for beginners to advanced algebra for more advanced students. Students can choose the level of difficulty that suits them best based on their confidence and proficiency in the subject. Educators promote self-regulation and autonomy in learning by providing students with choices and opportunities to assess their own learning progress. Students learn to set goals, monitor their performance, and adjust their strategies accordingly, leading to increased motivation, engagement, and academic success [16].

The effectiveness of teaching science can be negatively impacted by several issues that creative pedagogies can resolve [17]. Students better comprehend the ideas in the frequently abstract natural scientific textbooks by using interactive technologies, straightforward experiments, or visualizations as part of creative pedagogies [18]. Students can engage with natural science subjects meaningfully by incorporating artistic components into science instruction [19]. A learning strategy known as “creative pedagogy” strongly emphasizes adopting methods and techniques to encourage students' creativity during the educational process. The three components of creative pedagogy—creative teaching, teaching for creativity, and creative learning—are taken from the study findings of Lin [20]. The method a teacher uses to present educational materials that have enhanced and sparked students' imaginations is referred to as creative teaching. Teaching for creativity has brought attention to how important it is for teachers to support their students' creative growth by using methods that foster critical, analytical, and creative thinking. In the meantime, creative learning has focused on creating a setting in the classroom where students can explore new concepts and come up with creative solutions [20]. While there is much promise for students and teachers to improve learning effectiveness through creative pedagogy, some researchers have noted that its implementation has been fraught with difficulties, including shifting learning standards to a more flexible and creative approach. However, these difficulties have been surmounted with the correct assistance and cooperative efforts from curriculum developers, educational institutions, and teachers [21].

The pedagogical foundations of the differentiation approach in education stem from the belief that every student has unique learning needs, interests, and abilities. This approach acknowledges that a one-size-fits-all teaching method is ineffective for reaching all students and maximizing their learning potential. Instead, differentiation aims to tailor instruction to meet the diverse needs of learners within a classroom.

Here are some vital pedagogical foundations of the differentiation approach:

*Student-Centered Learning*: Differentiation places the student at the center of the learning process. It recognizes that learners come from diverse backgrounds and possess different learning styles, preferences, and abilities. Therefore, instruction is designed to accommodate these differences and empower students to take ownership of their learning.
*Flexible Instruction:* Teachers employing differentiation understand that not all students will master content at the same pace or through the same methods. They use a variety of instructional strategies, materials, and assessments to address students' individual needs. This might include modifying content, adjusting the pace of instruction, or providing alternative assignments.
*Assessment for Learning:* Assessment is integral to the differentiation approach. Teachers continuously assess student progress to determine their strengths, weaknesses, and areas for growth. This ongoing assessment informs instructional decisions, allowing teachers to tailor their teaching to effectively address individual student needs.
*Flexible Grouping:* In differentiated instruction, grouping strategies are used strategically to support collaborative learning and individualized instruction. Teachers may group students based on readiness, interests, or learning styles, and these groups may change frequently based on specific learning goals and activities.
*Respect for Diversity*: Differentiation promotes a culture of inclusivity and respect for diversity within the classroom. It values each student's unique background, experiences, and perspectives, fostering a supportive learning environment where all students feel valued and included.
*Teacher Expertise and Reflection:* Implementing differentiation requires skilled teachers who can effectively assess student needs, plan and deliver differentiated instruction, and reflect on its effectiveness. Continuous professional development and reflection are essential for teachers to refine their differentiation practices and meet the evolving needs of their students.

By embracing these pedagogical foundations, educators can create more inclusive, engaging, and effective learning environments that support the success of all students.

Teaching and learning are vital educational processes that prepare young individuals for the greater good of society. This greater good is determined not solely by the needs of the youth, but by the collective needs of humanity. It necessitates that all young people, regardless of their individual abilities, disabilities, race, language, or religion, have access to the highest quality educational opportunities available in their time. To achieve this goal, all forms of differentiation must be employed to ensure that every student receives optimal educational opportunities. Differentiation in teaching is a methodological framework designed to cater to individual learning needs and optimize students' academic experiences, potentially resulting in positive learning outcomes. Once instruction and curriculum are differentiated, various forms of assessment can be developed to accommodate the diverse needs of students. Given that classrooms consist of students with varying backgrounds and learning profiles, teachers must become part of the solution by differentiating their teaching methods and instructional programs. Through this approach, teachers can create assessments that accurately gauge students' comprehension of the taught concepts at an appropriate level [22].

Teaching and learning are increasingly challenging due to diverse student abilities and disabilities. However, there are differentiation processes that, if implemented, can offer enhanced opportunities for students to engage in their learning actively. It's crucial to recognize that ensuring every child achieves their personal best in the classroom is not just an educational but also an ethical imperative. Evidence supports the importance of differentiation in pedagogical approaches for fostering the well-being and success of students in their learning journey. This involves tailoring instructions, content, curriculum, and assessments to meet individual student needs. Such approaches enable students to persevere in their learning, develop a genuine interest in school, collaborate effectively with teachers, and cultivate a desire to gain a deep understanding of the subject matter. For these approaches to resonate with students, schools must develop engaging and relevant curricula that motivate all learners, even in challenging circumstances [23]. Moreover, the curriculum should be aligned with equally customized assessments to accommodate diverse abilities. By differentiating both the curriculum and assessments, students can thrive and experience a sense of fulfillment in their academic endeavors [24].

### Materials

K.A. Jirkova studied differentiated approaches and their implementation in foreign language learning in schools. Her approach began with determining criteria for differentiation, such as ability, knowledge, skills, learning capacity, training, creative abilities, and memory. She utilized previously acquired abilities and skills in her research. The next step involved selecting educational materials (tests, assignments) to group students. Students struggling with tasks were placed in weaker groups. Differentiation in English language teaching considered various implementation strategies:

- Differentiated complexity of the task and the same conditions for its fulfillment for everyone;
- Differentiated task complexity and differentiated conditions for its implementation;
- The exact complexity of the task for all students and differentiated specific conditions for its implementation;
- The task is of the same complexity for all students and has the same fulfillment conditions.

The most significant interest in implementing a differentiated approach is internal division (differentiation), which is pedagogy and psychology. Classwork has three types (forms): frontal, group, and independent. In our scientific experience, the differentiated approach was implemented the following way.

First of all, we determined by what criteria (ability, knowledge, skills, learning ability, training, level of creative abilities, memory) differentiation of training will be carried out. In our research, we relied on the abilities obtained from earlier knowledge and skills. The next stage was the selection of educational materials (tests, assignments) that allowed students to be divided into groups. Then, the students were diagnosed: those who had difficulties completing tasks were transferred to a weaker group.

In the experiment, students worked on tasks of the same complexity (text) but under different conditions, focusing on the reading skills of 3rd-grade students. The primary goal was to monitor reading skill development and text comprehension, requiring students to complete related tasks [25]

Similarly, Y.V. Valtseva explored a differentiated approach to foreign language teaching. Despite its use in various subjects, it still needs to be utilized in foreign language education due to the subject's practical focus and students' perception of it as secondary. Teachers, therefore, seek ways to enhance classroom learning effectiveness.

Given that students' abilities, learning pace, interests, and needs vary, teachers often cater to the average student, neglecting both the “strong” and the “weak.” Differentiated learning addresses individual abilities and needs by specifying goals, content, and teaching methods and necessitates diverse learning strategies. Physiologists believe integrating differentiation can reduce teaching load and positively impact students' health.

Effective differentiation can:

1) prevent gaps in the knowledge, skills, and abilities of students; equalize the level of preparation of the entire class;
2) develop the abilities and interests of students;
3) improve the quality of knowledge;
4) make more efficient use of everyone's study time;
5) involve all students in active, intense mental activity;
6) eliminate the gap between frontal teaching methods and the individual nature of knowledge [26].

Differentiation within the classroom-lesson system can lead to a more personalized educational process. Individualized educational work can take three forms: frontal, group, and independent. In frontal work, teachers present texts of varying complexity, simplify and complicate material, and encourage students to engage beyond the curriculum. Group work activates students by allowing them to express opinions and solve problems according to their interests and abilities, with groups formed based on performance levels. Independent work requires mental effort and self-directed learning, with students overcoming feasible but challenging tasks.

Differentiated learning also involves varying the conditions for task completion, such as teacher assistance, time allocation, thought processes, and monitoring forms [27].

### Method

In this review, we searched two concepts of differentiation approach in education. They are differentiated learning and differentiation approach, and their comparisons in the primary class.

### Result

Jirkova's research highlighted the importance and effectiveness of differentiated approaches in foreign language learning, particularly in developing reading skills and comprehension among young learners.

The research emphasizes the significance of differentiated learning, which involves understanding student traits, grouping them accordingly, and providing tasks suited to their abilities to enhance cognitive and moral growth.

Her findings and results show that by assessing students' abilities, knowledge, skills, learning capacity, training, creative abilities, and memory, Jirkova established clear criteria for differentiation. This initial step ensured students were grouped effectively based on their specific needs and capabilities. Educational materials such as tests and assignments were carefully selected to facilitate grouping. Students who struggled with tasks were placed in weaker groups, ensuring they received appropriate support and instruction tailored to their level. The experiment involved 3rd-grade students working on tasks of the same complexity (a text) but under different conditions. This approach aimed to monitor the development of reading skills and comprehension. The primary goal of the experiment was to observe the progression of reading skills and the ability to understand the text's central idea. The tasks designed for this purpose required students to demonstrate their comprehension through various related activities (Table 1).

**Table 1:** Jirkova's experiment findings based on differentiation approach.

Area	Details
Research Focus	Differentiated approaches in developing reading skills and comprehension in young learners
Purpose of Differentiation	Enhance cognitive and moral growth by tailoring instruction to individual student abilities
Differentiation Criteria	Students assessed based on abilities, knowledge, skills, learning capacity, creative abilities, and memory
Grouping Approach	- Students grouped by specific needs and abilities - Weaker students placed in groups for targeted support
Instructional Materials	Carefully selected tests and assignments to facilitate effective grouping
Experiment Design	- Conducted with 3rd-grade students - All students worked on the same task (a text) under varied conditions
Objective of the Experiment	- Monitor development of reading skills and comprehension - Track ability to understand main ideas in text
Task Complexity & Conditions	Tasks of uniform complexity but varied learning conditions to match individual student needs
Key Findings	- Grouping based on ability allows targeted instruction - Enhanced engagement and outcomes through task and support differentiation
Reading Skill Improvement	Noticeable improvement in reading skills and comprehension, demonstrating the effectiveness of differentiation
Student Engagement	Differentiated tasks-maintained interest and motivation by aligning with students' abilities and needs

Similar to this, Valtseva's research demonstrated that differentiated learning can significantly improve the effectiveness of foreign language teaching, making it a valuable approach for addressing students' diverse needs.

The research findings highlight the effectiveness of varied instructional methods, including frontal presentations and group work, which accommodate individual differences and encourage active student engagement through differentiated tasks and conditions.

Differentiated learning is less commonly used in foreign language education despite its effectiveness in other subjects. This is due to the subject's practical focus and students' perception of it as less important. Teachers often target the average student, which leads to neglecting both high-performing (“strong”) and low-performing (“weak”) students. This creates a need for differentiated learning to address all students' varying abilities, learning paces, interests, and needs (Table 2).

**Table 2:** Valtseva's experiment findings based on differentiated learning.

Area	Details
Research Focus	Effectiveness of differentiated learning in foreign language education
Purpose of Differentiation	Address diverse student needs, learning paces, and abilities to improve overall learning outcomes
Key Instructional Methods	- Frontal presentations - Group work - Varied instructional methods to accommodate individual differences
Differentiated Tasks	Tailored tasks and conditions to encourage active engagement
Benefits of Differentiation	- Improved effectiveness in language learning - Enhanced student motivation and participation
Experiment Design	- Conducted with 3rd-grade students - All students worked on the same task (a text) under varied conditions
Challenges in Foreign Language Education	- Differentiated learning is underused in language subjects - Practical focus and lower perceived importance by students
Task Complexity & Conditions	Tasks of uniform complexity but varied learning conditions to match individual student needs
Common Teaching Issue	Standardized teaching targets the average student, neglecting high (“strong”) and low-performing (“weak”) students
Need for Differentiation	Address the needs of all students with varied abilities and interests to prevent any group from being overlooked

## Discussion

It is not necessary for the teacher to be directly involved in independent work. It takes mental effort to finish the assignment. If students don't acquire effective procedures for differentiated work, the teacher won't get good results.

Through the use of organizational structures, differentiated learning allows each student to work at their own pace, completing tasks that are achievable but still sufficiently difficult. Not only should the tasks be different in difficulty under the differentiated approach, but the methods of tracking task completion should also be modified, as should the circumstances under which they are completed (e.g., different amounts of teacher support for strong and weaker students, different time allotments for task completion and response reflection).

**Fig. 1:**
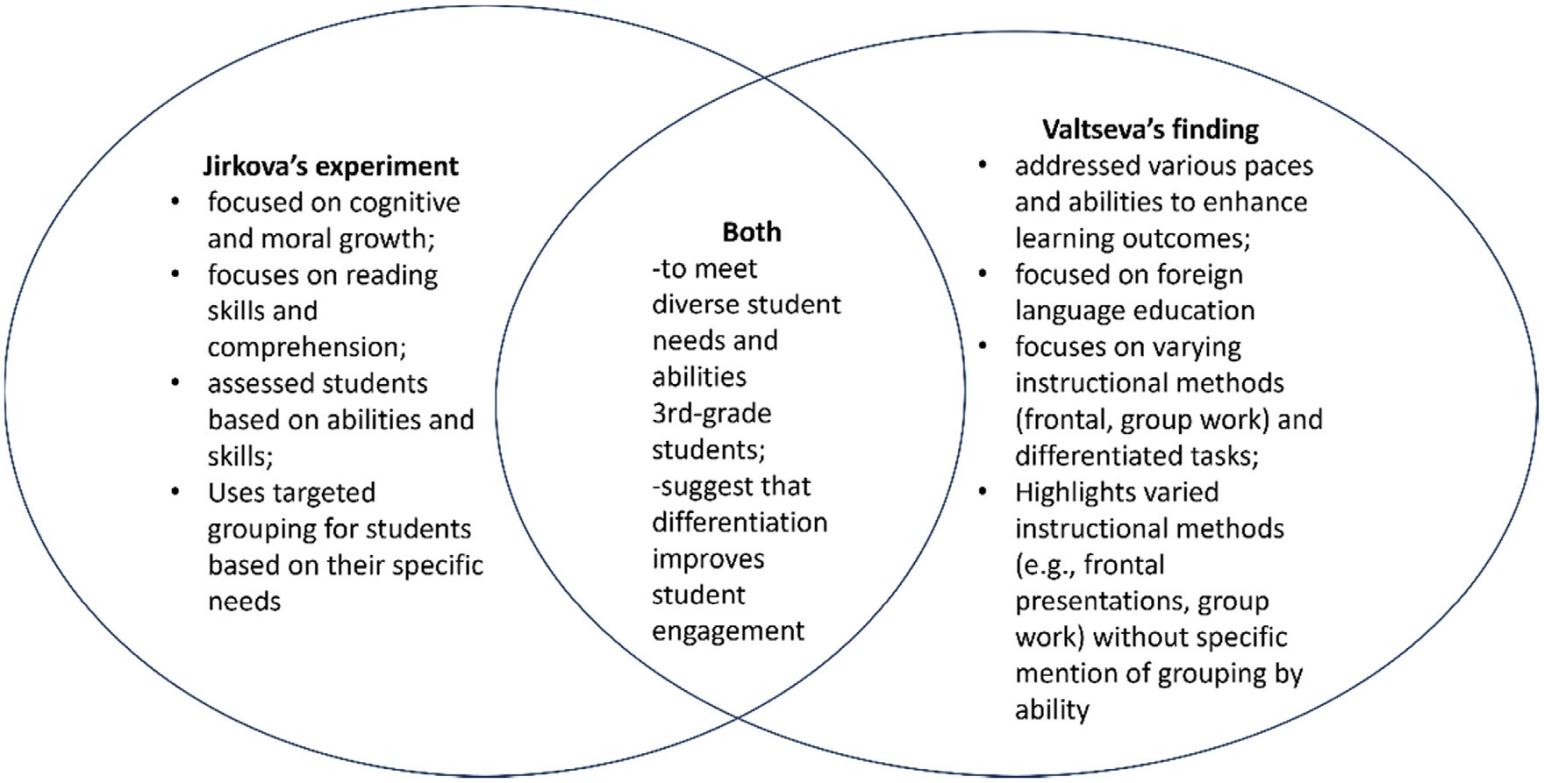
Similarities and differences between Jirkova's and Valtseva's experiment.

From Fig. 1, we can see the similarities and differences between Jirkova's and Valtseva's work. Jirkova used differentiation approach in the class, where the different task is based on the multiple intelligence of the students and also focused on the diverse needs of students, whereas Valtseva's findings is based on differentiated learning, which all students worked on the same task (a text) under varied conditions. In addition, the teacher standardized teaching targets the average student, neglecting high (“strong”) and low-performing (“weak”) students.

By considering the characteristics and circumstances of each student, differentiated learning methods can be used to address heterogeneity in the classroom. Repetition, specialized exercise routines, and complimentary or modified content are examples of diversified instruction strategies [28]. These approaches encourage the development of competence at various levels of success. Additionally, students increase the efficacy of differentiated education. However, differentiated instruction enhances learning outcomes, student performance, and their ability to critically reflect on their own work, it is as beneficial when implemented in junior high school classrooms [29].

Finally, for teaching and learning to be successful, the learning environment must be changed. Teachers are specifically expected to modify the classroom setting in order to successfully include every student in the learning activities. This also includes the capacity to employ culturally responsive teaching pedagogy, encourage student collaboration, guarantee resource availability, and employ appropriate communication tones with students [30].

## Conclusion

K.A. Jirkova and Y.V. Valtseva's studies highlight the effectiveness of differentiated approaches in foreign language education. Jirkova's research focused on developing criteria for differentiation and grouping students based on their abilities, which enhanced reading skills and text comprehension among 3rd-grade students. Valtseva's exploration of differentiated learning emphasized its potential to address individual student needs, improve engagement, and enhance overall learning outcomes. Both studies demonstrated that differentiated teaching strategies can prevent knowledge gaps, develop student abilities and interests, and promote a more personalized and effective educational process. Integrating differentiated approaches in foreign language teaching can reduce teaching load, positively impact student health, and better cater to the diverse needs of learners.

## Ethics statements

In the research process, it can be seen that the differentiation approach was used among students in 2013 and 2014, but this approach was not used in schools experimentally or in the teaching process, as instead of debating whether or not to differentiate instruction, educators should concentrate on how to do it and differentiated learning supports the claim that teacher preparation is lacking since it shows that teachers are not consistently implementing in their classrooms [31]. Differentiating instruction is more challenging in practice than it appears, as highlighted by Wan (2016). Teachers were unable to accommodate the diversity of learners with the same ease because they lacked sufficient experience with differentiation tactics. Teachers struggle to understand how differentiated instruction should be applied in their classrooms, despite the fact that the concept is widely recognized [32]. By the 2020s, a differentiated approach has proven to be beneficial for students with diverse abilities. Differentiated learning, which is more successful than standard classroom approaches at boosting student enthusiasm and comprehension in science classes, is taught in elementary school classrooms with an emphasis on student learning styles [33].

## CRediT authorship contribution statement

**Nigora Goyibova:** Conceptualization, Methodology, Investigation, Validation, Software. **Narzulla Muslimov:** Resources, Writing – review & editing, Methodology. **Gulnoza Sabirova:** Visualization. **Nargiza Kadirova:** Writing – original draft. **Barnoxon Samatova:** Supervision, Software.
